# A comprehensive review on diabetic cardiomyopathy (DCM): histological spectrum, diagnosis, pathogenesis, and management with conventional treatments and natural compounds

**DOI:** 10.1007/s00210-025-03980-9

**Published:** 2025-03-18

**Authors:** Ahmed R. Abdullah, Mahmoud A. Seliem, Emad Gamil Khidr, Ayah M. Sobhy, Riham A. El-Shiekh, Mohamed S. Abd El Hafeez, Ahmed A. El-Husseiny

**Affiliations:** 1 Biochemistry and Molecular Biology Department, Faculty of Pharmacy, Al-Azhar University, Cairo, 11231 Egypt; 2 Department of Biochemistry, Faculty of Pharmacy, Ahram Canadian University, 6Th of October City, Giza, Egypt; 3 Pharmacognosy Department, Faculty of Pharmacy, Badr University in Assiut, Assiut, Egypt; 4 Department of Pharmacognosy, Faculty of Pharmacy, Cairo University, Kasr El-Aini Street, Cairo, 11562 Egypt; 5 Department of Pharmacy, Kut University College, Al Kut, Wasit, 52001 Iraq; 6 Department of Pharmacognosy, Faculty of Pharmacy, Egyptian Russian University, Cairo-Suez Road, Badr City, 11829 Egypt; 7 Department of Biochemistry, Faculty of Pharmacy, Egyptian Russian University, Badr City, Cairo 11829 Egypt

**Keywords:** Diabetic cardiomyopathy (DCM), Molecular and cellular pathways, Diabetes complications, Cardiovascular diseases, Natural products

## Abstract

Diabetic complications are among the most pressing health issues currently. Cardiovascular problems, particularly diabetic cardiomyopathy (DCM), are responsible for almost 80% of diabetic deaths. Because of the increasing prevalence of diabetes and the increased threat of death from its consequences, researchers are searching for new pharmaceutical targets to delay or cure it. Currently, there are a few medicines available for the treatment of DCM, some of which have serious side effects. To address this issue, researchers are focusing on natural products. Thus, in this review, we discuss the prevalence, incidence, risk factors, histological spectrum, diagnosis, pathogenic pathways of DCM, genetic and epigenetic mechanisms involved in DCM, the current treatments, and the beneficial effects of natural product–based therapeutics. Natural treatments range from single doses to continuous regimens lasting weeks or months. Flavonoids are the largest class of natural compounds reported for the treatment of DCM. Natural regimens may cover the way for new treatment strategies for DCM for being multi-target agents in the treatment of DCM, with the ability to play a variety of functions via distinct signaling pathways.

## Introduction

Patients with type 2 diabetes (T2DM) have elevated blood pressure (BP), which contributes to the development of cardiovascular diseases (CVD), a leading cause of morbidity and mortality worldwide, accounting for over 70% of fatalities (Kohli et al. 2013). Diabetic cardiomyopathy (DCM) grows as myocardial dysfunction in the absence of hypertension and coronary artery disease (Aneja et al. 2008). Hyperglycemia is critical to its etiology, triggering a cascade of maladaptive stimuli that result in collagen deposition and cardiac fibrosis. These progressions have been proposed to be responsible for altered myocardial relaxation description and are visible as diastolic dysfunction on imaging (Bugger and Abel 2014). DCM, the most common disease caused by diabetes complications, is distinguished by atypical myocardial structure and function. Symptoms include increased free radical generation, lipid peroxidation, lipid accumulation, energy deficit, mitochondrial dysfunction, advanced glycation end-product (AGEs), activation of protein kinase C isoforms, imbalance in ATP/O_2_^−^ consumption ratio, and activation of peroxisome proliferator–activated receptors (Rubler et al. 1972). Cardiac hypertrophy is recognized as a key feature of DCM. Currently, DCM treatment follows standard heart failure management guidelines, but no specific therapies have received approval. This highlights the urgent need for further research to deepen our understanding of this complex syndrome (Parim et al. 2019). To overcome these challenges, searching for cost-effective, advanced, unique treatments becomes the most critical responsibility in providing long-term relief to DM patients. Natural products remain a valuable source of scaffolds with a wide variety of structural diversity and bioactivity, which have the potential to be created directly or utilized as starting points for optimizing novel medications (Atanasov et al. 2021). Natural products have recently been proven to be successful as anti-diabetic medicines, both in vitro and in vivo, as well as clinical trials (Alam et al. 2018; Jugran et al. 2021). As a result, the purpose of this review is to raise awareness about the present state of DCM, its treatments and side effects, and the benefits of using natural compounds to combat it.

## The prevalence and the incidence of DCM

It has been established that diabetes mellitus (DM) is linked to CVD, and the prevalence of DM has been rising gradually globally. Over 10% of adults worldwide are diagnosed with DM (Marx et al. 2023). The significant burden of DCM is a result of this high prevalence. People who have had diabetes for a long time, have poor glycemic status, and have other cardiovascular risk factors are much more likely to develop DCM. Up to 30% of diabetic patients have been shown to develop DCM, and the prevalence rises (12 to 22%) with the course of DM (after 10 years of the disease period) (Boudina and Abel 2010).

According to clinical investigations, the prevalence of DM-induced heart failure extends from 19 to 26% (Jia et al. 2018a). Men and women with T2DM are very likely to have left ventricular diastolic dysfunction, whereas the entire people and type 2 diabetic patients under hospitalization had a pooled prevalence of about 35% and 48% of left ventricular diastolic dysfunction, respectively (Bouthoorn et al. 2018). The overall incidence of type 1 diabetic patients associated with heart failure was 5.8%, and the risk of heart failure is three times greater in T1DM patients than in controls (Haji et al. 2023).

The elevation of glycated hemoglobin (HbA1c) by 1% in type 2 diabetic patients was reported to raise the heart failure susceptibility by 30%, while the heart failure risk rose by only 8% in patients with type 1 diabetes for each 1% increase in HbA1c, despite additional risk factors such obesity, hypertension, smoking, coronary heart disease, and dyslipidemia. This suggests that rated elevations in glycemic status are a potent inducer of diabetes-mediated heart failure (Avagimyan et al. 2024).

## Risk factors of DCM

The rising rate of heart failure in patients with diabetes can be clarified by the similarity between risk factors that affect diabetic patients and general individuals. Hypertension and dyslipidemia are the most frequent risk factors for congestive heart failure (CHF), and they are both much more common in people with diabetes. Other factors, such as reinfarction and prior infarction, have greater susceptibility than dyslipidemia and hypertension to developing heart failure. The development and worsening of heart failure are influenced by coronary atherosclerosis, which is raised in diabetes. The incidence of CHF is increased by the interaction of these variables more than by any one of them alone (Wang et al. 2006).

In addition, diabetes is linked to several complications, including peripheral insulin resistance, endothelial dysfunction, autonomic neuropathy, hyperglycemia, and irregular cardiac fuel consumption, all of which increase the likelihood of cardiomyopathy (Sun et al. 2019). The mortality susceptibility was raised by 53%, 16%, and 9% in patients with blood glucose concentrations of ≥ 200, 140 to 200, and 110 to 140 mg/dL, respectively, in comparison to the controls without diabetes with glucose levels < 110 mg/dL. Interestingly, blood glucose levels and long-term mortality in heart failure were linearly correlated, even in individuals who were never diagnosed with diabetes mellitus. Nevertheless, the elevation of blood glucose to more than 200 mg/dL in diabetics showed an elevated risk of death (Sun et al. 2019). Extended glycemic control had been revealed to be associated with the likelihood of heart failure, which was proved by the declining heart failure risk by 16% as a result of decreasing HbA1c by 1% (Bahrami et al. 2008).

## Histological spectrum of DCM

**Fig. 1:**
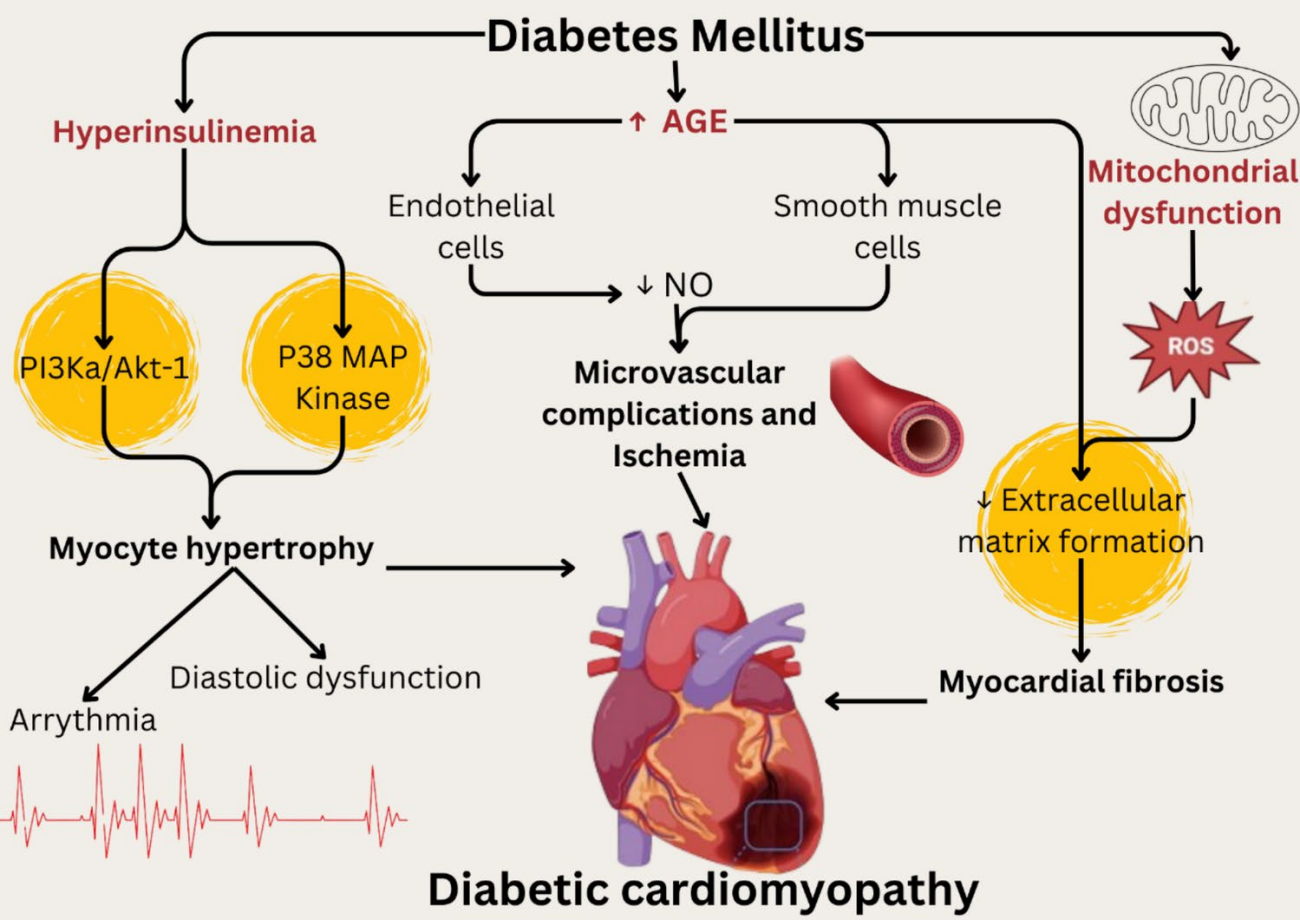
Connections between histological abnormalities, their causes, and diabetic cardiomyopathy

A complex and multifactorial combination of metabolic, hemodynamic, and genetic changes highlights the histological abnormalities linked to DCM (Gigli et al. 2024). Numerous significant histological characteristics have been found in diabetic hearts (Fig. 1).

### Myocardial fibrosis

A notable histological observation in DCM is myocardial fibrosis, which arises from an imbalance between the formation and breakdown of the extracellular matrix (Lunde et al. 2024). The development of DCM is also linked to elevated AGE production in diabetic tissue (Bodiga et al. 2014). AGEs induce fibrosis and hamper cardiac relaxation by forming crosslinks with extracellular matrix proteins. The cardiac tissue becomes stiffer due to excessive collagen deposition, which reduces its contractility (Cowling et al. 2019). This fibrosis, which can be perivascular (around blood vessels) or interstitial (between myocardial cells), is thought to be the main cause of diastolic dysfunction in DCM (Nikolajević Starčević et al. 2019). According to several studies, fibrosis has been linked in diabetic patients to decreased cardiac activity and a higher likelihood of heart failure (Segura et al. 2014; Russo and Frangogiannis 2016).

### Myocyte hypertrophy

Myocyte hypertrophy is frequently seen in hearts with diabetes. Initially, it was believed that this adaptation was a compensatory mechanism to maintain cardiac output (Swynghedauw 1999). Nevertheless, massive hypertrophy eventually raises the potential of arrhythmias and causes diastolic dysfunction (Gupta et al. 2007). Besides, it has been attributed to the consequence of direct insulin signaling via p38 MAP kinase and PI3Ka/Akt-1 cascade despite cellular insulin resistance. Moreover, chronic hyperinsulinemia indirectly increases cardiac Akt-1 activation by activating Ca^2+^-calmodulin-dependent kinase through β2-adrenergic receptors and stimulating protein kinase A (PKA) mediated by the sympathetic nervous system (Kanamori et al. 2021). Furthermore, myocyte atrophy might happen later, which would lead to heart failure and systolic dysfunction (Falcão-Pires and Leite-Moreira 2012; Goyal and Mehta 2013).

### Inflammation and oxidative stress

The development of DCM is significantly impacted by chronic low-grade inflammation and elevated oxidative stress. The cardiac interstitial exhibits a high level of macrophage infiltration in diabetes individuals (Jin et al. 2019). The myocardial buildup of AGEs can set off inflammatory reactions that activate pro-fibrotic pathways (Frati et al. 2017). Additionally, myocardial injury and fibrosis are triggered by reactive oxygen species (ROS), which also compromise myocytes and the extracellular matrix (Ansley and Wang 2013). One of the characteristics of DCM is mitochondrial dysfunction. Mitochondrial damage, which hinders ATP synthesis and worsens oxidative stress, is frequently seen in diabetic hearts. Increased cell apoptosis is another effect of this mitochondrial dysfunction, which exacerbates myocardial damage and remodeling (Tsutsui et al. 2009).

### Vascular alterations

Coronary microvascular function alterations are also linked to DCM (Picchi et al. 2010). Through AGE receptors (RAGEs) on a range of cells, such as macrophages, endothelial cells, and smooth muscle cells, the increased AGEs in DCM reduce the generation of nitric oxide (NO), which leads to the emergence of microvascular complications and eventually aggravates ischaemic injury and myocardial remodeling (Koulis et al. 2015; Wang et al. 2024).

## Diagnosis of DCM

The DCM is clinically diagnosed as ventricular dysfunction in diabetes cases without coronary atherosclerosis or hypertension (Lorenzo-Almorós et al. 2017). Diastolic dysfunction, systolic dysfunction, and ultimately clinical heart failure due to an unknown etiology are the hallmarks of its clinical course (Falcão-Pires and Leite-Moreira 2012). Although the early diagnosis of DCM is essential for controlling the development of heart failure, it is nevertheless a challenge due to the lack of precise diagnostic standards. The diagnosis of DCM has been enhanced by advances in imaging technologies, including cardiac MRI, echocardiography, and the use of biomarkers (Table 1); however, the histological investigation may still be necessary for a conclusive diagnosis (Huo et al. 2023a).

**Table 1:** Different approaches for recognition of the DCM, ranging from clinical to sophisticated imaging techniques

Diagnostic tools	Purpose	Advantages	Disadvantages	Ref
Clinical characteristics	As the DCM worsens, patients may have symptoms like fatigue, dyspnoea, and decreased tolerance to physical activity	Patients with chronic diabetes, particularly those with poorly managed blood glucose levels and concomitant conditions like hypertension, are more likely to exhibit clinical suspicion	Early DCM is frequently asymptomatic	Boord et al. (2001), Gulsin et al. (2019)
Heart rate variability (HRV) check	It is a reliable metric for evaluating cardiac autonomic neuropathy and can assess the risk of cardiovascular problems and track the course of the disease	It aids in detecting autonomic dysfunction before anatomical alterations in the heart and predicting arrhythmia. Also, it is simple and cost-effective	Stress, drug intake, and physical exercise are some of the variables that might affect HRV Limited specificity	Metelka et al. (2018)
Electrocardiogram	It reveals abnormalities in the electrical impulses of the heart utilizing electrodes	It can spot irregularities before symptoms of heart failure appear It can detect arrhythmia, cardiac autonomic dysfunction, and left ventricular hypertrophy	Limited sensitivity and specificity The cardiac muscles cannot be directly imaged by ECG	Bildirici et al. (2010)
Echocardiography	It provides real-time images of your heart using sound waves. Relevant metrics like diastolic dysfunction left ventricular ejection fraction, and left ventricular hypertrophy are frequently evaluated	It is regarded as the most popular method for diagnosing DCM. Also, it enables precise and reproducible diagnostic and prognostic information for DM cases. Notably, diastolic dysfunction is an early indicator of DCM that can be seen even when systolic dysfunction is absent. Diastolic filling pressures and the velocity of blood flow across the mitral valve can be measured using Doppler approaches, which aid in the early detection of cardiac abnormalities linked to DCM	Its application in DM patients is still disregarded, leading to the development of DM-related heart failure in several patients	Galderisi (2006), Ernande et al. (2011), Dragulescu et al. (2013), Lorenzo-Almorós et al. (2017), Urlic et al. (2023)
Cardiac magnetic resonance imaging (MRI)	By employing late gadolinium enhancement (LGE) imaging, cardiac MRI can assess myocardial fibrosis, which aids in distinguishing DCM from other heart failure causes	It permits an accurate evaluation of ventricular anatomy, function, and wall motion. Besides, it is safe for follow-up investigations	Compared to other imaging approaches, cardiac MRI is more costly, time-consuming, and limited accessibility Also, cardiac MRI is less useful for imaging dynamic cardiac events in actual time than echocardiography	Ambale-Venkatesh and Lima (2015)
Multi-slice computed tomography (MsCT)	It employs volumetric approaches to provide ventricular function data	Pixel-by-pixel computations of end-systolic and diastolic volumes are made utilizing the automated program that organizes and reconstructs various heart rotations. MsCT has been shown as a potential tool for ischaemic heart disorder by evaluating atherosclerosis and coronary artery calcification	Due to side effects, utilization of contrast media, and radiation exposure, DCM diagnosis may need to be done using alternative methods	Lorenzo-Almorós et al. (2017)
Nuclear imaging	These methods rely on cardiac autonomic dysfunction, which is frequently observed in the early stages of diabetes	It renders feasible to assess cardiac metabolism and evaluate molecular imaging by enabling the recognition of low-density activities	Their scans are not infallible despite their propensity for precision and accuracy Radiation exposure and high cost The length of time required to figure out the imagery	Adel and Chen (2024)
Serum biomarkers	Cardiac troponin can evaluate left ventricular dysfunction. Also, elevated B-type natriuretic peptide (BNP) or N-terminal proBNP indicates raised cardiac stress and heart failure, while CRP can assess cardiac inflammation	Their evaluations are non-invasive, cost-effective, and accessible. Besides, they can be utilized in monitoring DCM progression to assess the long-term efficacy of therapeutic approaches	Due to a lack of specificity, no single biomarker is generally recognized as the gold standard For DCM diagnosis	Haller et al. (2023), Neves et al. (2024)
Endomyocardial Biopsy	It renders feasible to directly evaluate cardiac fibrosis, myocyte enlargement, and other histological alterations linked to DCM	It can offer conclusive histological evidence of DCM	It is not frequently used in clinical practice since it is invasive	Frustaci et al. (2002), Khan et al. (2017)
Exercise stress test	It is frequently utilized to assess cardiac function for patients with diabetes and suspected DCM	It indicates that symptoms are prompted by DCM rather than coronary artery obstructions It is inexpensive	It is limited to detecting early DCM It is difficult to perform for elderly patients	Vanzetto et al. (1999)

## The pathogenic pathways of DCM

**Fig. 2:**
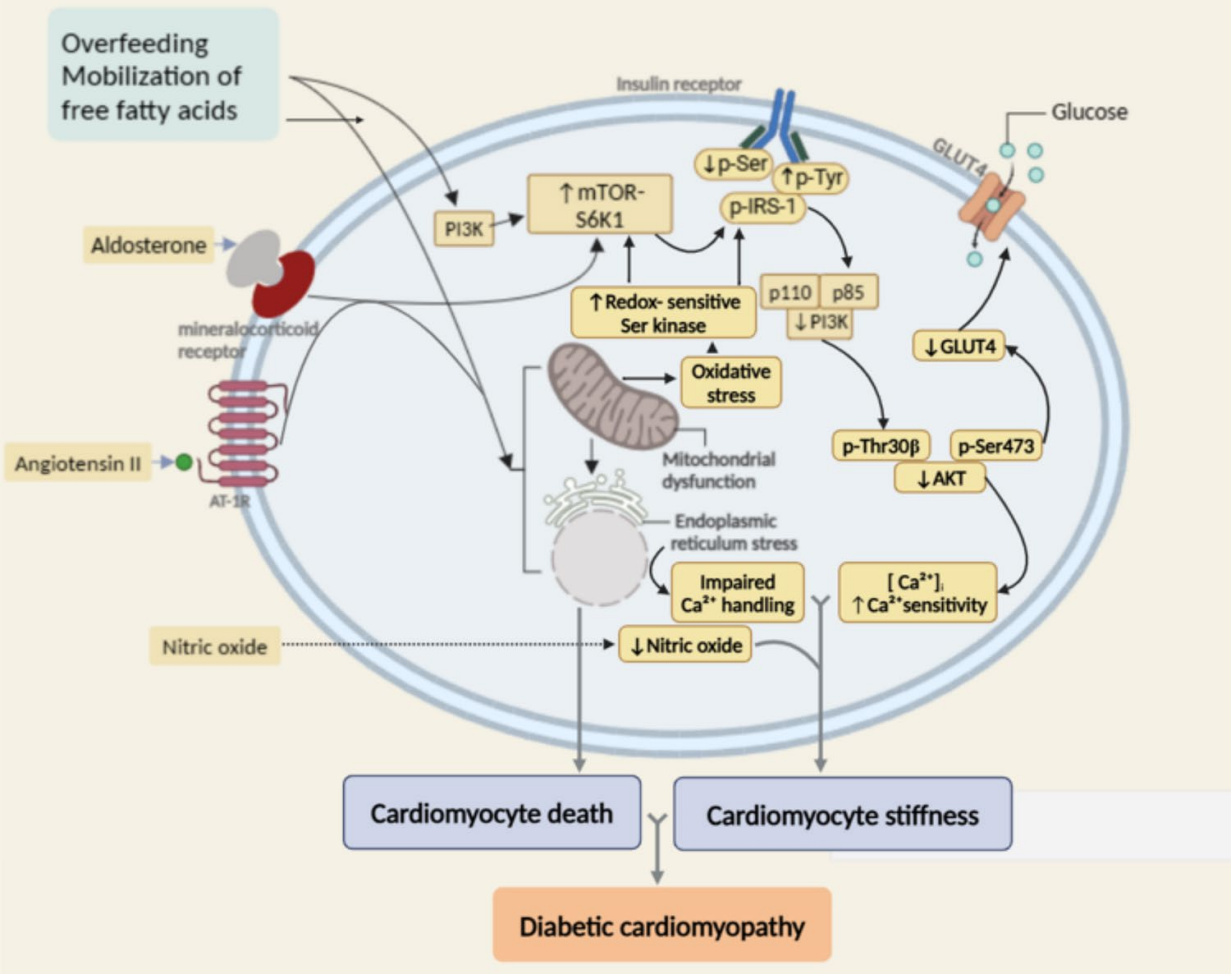
The pathophysiological mechanisms of diabetic cardiomyopathy (DCM)

The pathways that emphasize the mechanisms underlying DCM are intricate and still unclear. (Huo et al. 2023b). Several factors have been identified as to the etiology of cardiomyopathy, with new ones being discovered every year. Among these are abnormal glucose metabolism with resistance to insulin, abnormalities of subcellular components, microvascular impairment, cardiac autonomic malfunction, dysregulation in the RAAS, metabolic disorders, inflammatory disorders, oxidative stress, unbalanced immune responses, and impaired Ca^2+^ handling (Fig. 2). Other factors include changes in gene regulation, insulin signaling, endoplasmic reticulum stress, mitochondrial dysfunction, neurohumoral activation, mitochondrial metabolism/utilization, and cardiac cell death (Marfella et al. 2021).

### Insulin resistance and abnormal glucose metabolism

DCM is triggered by persistent hyperglycemia, which raises gluconeogenesis and lowers glucose clearance (Jia et al. 2016). In DCM, the uptake of glucose is diminished, and its metabolism is unregulated as a result of the reduction in glucose transporters 1 and 4 (Fazakerley et al. 2009). In addition, hyperinsulinism and cardiac insulin resistance considerably minimize glucose consumption in patients with DCM (Jia et al. 2018b). Therefore, lipids deposit in cardiomyocytes, as well as lipid precursor metabolites, such as diacylglycerol, uncoupling protein-3, and ceramide, as a result of the oxidation of nonesterified fatty acids, as a source of energy for cardiac tissues. The overproduction of these substances impacts the myocardium’s energy supply, leading to myocardial dysfunction, cardiomyocyte necrosis, and myocardial fibrosis, ultimately resulting in abnormality in aberrant cardiac function and structure (Bugger and Abel 2014). Moreover, numerous investigations have verified that long-term hyperglycemia stimulates the production of reactive oxygen species through the electron transport chain. By directly driving glycosylation, suppressing phosphoglyceraldehyde dehydrogenase, and activating poly ADP-ribose polymerase, ROS inhibit the normal glycolytic route and trigger further detrimental cascade events, such as stimulation of protein kinase C and an increase in Glycation end-products (Burkart et al. 2007; Isfort et al. 2014). Consequently, glycation end-products, which are produced by the glycosylation of lipoproteins, amino acids, and lipids non-enzymatically and linked to sugars, prevent collagen breakdown and promote cross-linking between connective tissues and the protein of the extracellular matrix covalently; as a result, cardiac stiffness and abnormal diastolic relaxation lead to an increase in damage and fibrosis of myocardial tissues (Grubić Rotkvić et al. 2021).

### Elevation of reactive oxygen species

The excessive metabolism of glucose in hyperglycemia produces reactive oxygen species in mitochondria, which in turn induces oxidative stress. As a result, cell DNA damage and cardiomyocyte apoptosis are triggered by ROS-induced oxidative stress. For instance, the excessive production of superoxide in the respiratory chain of the mitochondria is linked to myocardial fibrosis by reducing the myocardium’s contractility (Al Hroob et al. 2019).

Excessive hyperglycemia has been demonstrated to induce NF-κB-mediated inflammatory signaling and suppress the antioxidant signaling of Nrf2 and Sirt1. Cardiac dysfunction and remodeling are exacerbated by the interaction of oxidative stress and inflammation, which raises the production of ROS and inflammatory molecules (Tang et al. 2022). In addition, the third gasotransmitter, hydrogen sulfide (H_2_S), may be essential to the cardiovascular system. H_2_S shortage worsened diabetic cardiomyopathy in mice’s hearts by aggravating mitochondrial damage, stimulating the NLRP3 inflammasome, boosting necroptosis, and increasing ROS formation (Gong et al. 2022).

### Alteration of calcium handling

The rate, intensity, and duration of myocyte contraction are largely influenced by the total concentration of calcium that enters the cytoplasm and the rate of calcium elimination from it. Thus, calcium handling is crucial to avoid abnormalities in cardiac function. A longer diastolic period of relaxation is revealed in diabetic cardiomyopathy because of an elevation in action potential driven by the inadequate control of Ca^2+^ by the transporters. (Lou et al. 2012). According to Guzik et al.’s (2020) investigation, type 1 and T2DM rat models differ in intracellular decay extension of Ca^2+^, raised intracellular resting Ca^2+^, postponed Ca^2+^ transients, impaired sarcoplasmic reticulum (SR) function of Ca^2+^ reuptake, diminished SR pumping efficiency due to AGE cross-linking to the SR Ca^2+^ ATPase pump, a reduction in SR Ca^2+^ load, and Ca^2+^ leakage from SR (Guzik et al. 2020). The shortage of protein expression and messenger RNA of SR calcium-ATPase (SERCA), along with an overproduction of phospholamban (responsible for lowering SERCA’s binding to calcium), is the main reason for reduced SR capacity (Yan et al. 2020). These alterations have been linked to ventricular diastolic dysfunction as well as cardiomyocyte contractility initiation, which are reported in the initial phases of diabetic cardiomyopathy (Jia et al. 2018a).

### Activation of RAAS

Although the condition is characterized by overproduction of salt and fluid, the cardiac tissue and systemic RAAS activation are noticed in hyperglycemia and insulin resistance. Angiotensin II and aldosterone levels rise when cardiac tissue RAAS is activated, which has a variety of impacts on cardiomyocytes. Angiotensin II levels have been demonstrated to be 3.4 times higher in patients with diabetes than in non-diabetics (Lee and Kim 2017). The stimulation of receptor signaling of mineralocorticoid in heart muscle tissue and the type 1 receptor of angiotensin II is enhanced by increased aldosterone and angiotensin II production. This triggers an adaptive proinflammatory immune reaction that results in elevated cytokine expression, oxidative stress and inflammation, leukocyte adhesion, and macrophage infiltration (Grubić Rotkvić et al. 2021). All of these responses collaborate to further activate several pro-fibrotic and growth signaling pathways, which in turn induce diastolic dysfunction, cardiomyocyte fibrosis, and occasionally even heart failure (Jia et al. 2018b).

Additionally, hyperglycemia-induced abnormal RAAS activation has been linked to elevated vascular resistance and arterial pressure. Furthermore, it has been proposed that disrupted mineralocorticoid receptor activation signaling and increased angiotensin II stimulate the target of the signal transduction mechanism of rapamycin-S6 kinase 1 in mammals, which in turn stimulates insulin resistance (Lee and Kim 2017).

### Mitochondrial dysfunction

Mitochondrial oxidative phosphorylation provides 90% of the ATP needed by the heart under normal circumstances (Jia et al. 2018a). However, free fatty acids (FFAs) are high in the diabetic myocardium due to greater adipose tissue lipolysis and insulin resistance, which trigger the utilization of FFAs as a source of energy instead of glucose. This change causes oxidative stress and disrupts regular oxidative phosphorylation (El Hadi et al. 2019). Rapid ROS accumulation results from the heart’s ability to neutralize ROS being reduced in diabetes patients (Jia et al. 2018a). Besides, ROS can disrupt the regular dynamics of the mitochondria, leading to fragmentation and disruption in mitochondrial function (Ritchie and Abel 2020). Moreover, hyperglycemia has a significant role in cardiac mitochondrial abnormalities, such as mitochondrial swelling and reduction of mitochondrial number (Joshi et al. 2014).

### Lipotoxicity

An excessive amount of fat that causes alterations to several metabolic mechanisms and ultimately leads to detrimental effects on adipose tissue and peripheral organs is known as lipotoxicity (Yazıcı and Sezer 2017). One of the primary causes of insulin resistance and altered pancreatic beta cell function is lipotoxicity. The detrimental effect of insulin on the adipose tissue and liver is mainly induced by elevated FFAs in patients with diabetes (Ritchie and Abel 2020). Excessive accumulation of higher FFAs causes lipotoxicity in the heart, beyond the normal capability for storage and oxidation, inducing several cardiac-related complications, such as cardiomyopathy. The reciprocal synchronization of a cluster of differentiation 36 (CD36) and glucose transporter type 4 (GLUT 4), which is in charge of fatty acid uptake, mainly leads to unusual deposition of FFAs in the heart (Quinaglia et al. 2019). This mechanism also explains why fatty acids have an advantage over glucose as a fuel for energy in the mitochondria. Lipotoxicity also disrupts the heart’s insulin signaling system and the regular physiological process of autophagy, which leads to structural and morphological changes as well as a decline in myocardial function. These abnormal modifications reduce the effectiveness of muscle fibers’ response to electrical stimulation and allow oxygen to enter the myocardium (Granéli et al. 2019).

The lipotoxic effect can also be initiated through non-oxidative phosphorylation of some fatty acids, which generates intermediates such as ceramides and diacylglycerol (DAG) (Ritchie and Abel 2020). These metabolites alter insulin signaling pathways, which in turn causes diabetic cardiomyopathy. For example, DAG inhibits insulin metabolic signaling and nitric oxide generation via regulating protein kinase C, which in turn affects glucose metabolism. Fatty acid- and sphingosine-based ceramides directly stimulate abnormal PKCs, causing them to become phosphorylated. As a result, metabolic insulin signaling of Akt is inhibited, and the translocation of GLUT4 is decreased (Jia et al. 2018b). Furthermore, these harmful substrates interfere with regular biological functions and cause apoptosis, mitochondrial malfunction, and cellular damage, which progressively leads to contractile failure and cardiac fibrosis (Lee and Kim 2017).

## The genetics involved in DCM

It was revealed that the phenotype of diabetic hearts is regulated by abnormalities in cardiac gene regulation, specifically miRNA and epigenetic processes (Mittal et al. 2021). Numerous genes of short non-coding RNA have been linked to the development of numerous diseases, including diabetic cardiomyopathy (Evangelista et al. 2019). Thus, it is important to shed light on many essential functions of these non-coding RNAs in diabetic cardiomyopathy.

### miRNA involvements in DCM

MicroRNAs are abundant throughout multiple cardiac cell types, including fibroblasts, endothelial cells, and cardiomyocytes (Das et al. 2018). There is evidence that miRNAs have an essential function in the control of a variety of pathological changes, including mitochondrial dysfunction, myocardial fibrosis, cardiac hypertrophy, epigenetic modifications, apoptosis, and oxidative stress, which play a major role in the etiology of diabetic cardiomyopathy (Zhang et al. 2019b). Furthermore, several miRNA alterations have been identified in the cardiac tissues of diabetic mice in comparison to non-diabetic ones. Huynh et al. (2014) reported fourteen miRNAs that are overexpressed in diabetic cardiomyopathy patients (Huynh et al. 2014). These genetic alterations either induce the development of diabetic cardiomyopathy or exacerbate its consequences (Ritchie and Abel 2020). Heart fibrosis and hypertrophy have been linked to some miRNAs. Heart hypertrophic signaling has been linked to anti-hypertrophic miRNAs, including miR-208a, miR-451, and miR-221, as well as anti-hypertrophic miRNAs, including miR-1, miR-378, miR-373, miR-181a, miR-23b, and miR-30c. In addition, it has been found that miR-214, miR-150, miR-199a, miR-125b, miR-29a, and miR-212 contribute to hypertrophic growth. In addition, miR-133a and miR-373 influence myocyte enhancer factor signaling, playing a crucial role in both p300 gene activation, which induces cardiac fibrosis, and the regulation of transcription of myocardial hypertrophy (Guo and Nair 2017). Diabetes patients have been reported to have downregulated miR15a/b, and cardiac fibrosis has been associated with fibrotic signaling of transforming growth factor receptor 1 and connective tissue growth factor (CTGF) (Zhang et al. 2019b).

miRNAs were reported to have impacts on the processes of autophagy, pyroptosis, and apoptosis, which are all implicated in the progression of diabetic cardiomyopathy. Along with 34 miR-30a, miR-133a, miR-30c, miR-21235, and 33 miR-221, which have been connected to the control of autophagy in the myocardium of diabetic patients, it has been reported that overexpression of 26 miR-206, miR-1, 25, 29 miR-378, miR-34a32, miR-30b, 3p31, 11 miR-195, 30 miR-483, 11 miR-195, 27 miR-144, 23 miR-08a, and 28 miR-320 supports apoptosis in the heart of diabetic patients (Zhou et al. 2019). Notably, some microRNAs, including miR-373, miR-1, miR-133a, and miR-378, are associated with the development of oxidative stress as it is downregulated under diabetic conditions (Evangelista et al. 2019).

Additionally, modulation in heart structure, angiogenic regulation, myocardial electrical remodeling, inflammatory reaction, and mitochondrial dysfunction have all been linked to miRNAs. Reduction of myeloid cell leukemia 1 expression (which is involved in cell survival) and structural damage to the heart has been associated with overexpression of miR-29 in a diabetic model (Zhang et al. 2019b). Moreover, the reduction of mitochondrial phosphate transport, which impacts ATP synthesis, is caused by the upregulation of miR-141. Furthermore, diabetes-related disruption of the negative regulatory pathway involving miR-200 and Zeb1 has been associated with the stimulation of inflammatory reactions in the cells of vascular smooth muscle. In addition, it has been discovered that miR-193 and miR-301a-5p actively contribute to the development of diabetic cardiomyopathy by affecting its downstream gene IGF2, which controls the voltage-gated potassium channel Kv4 (Zhang et al. 2019b). Thus, these results imply that the signal transduction pathways and distinct molecular processes in the myocardium that are implicated in the progression of diabetic cardiomyopathy are influenced by the down-expression and upregulation of particular kinds of miRNAs.

### lncRNA involvements in DCM

Numerous lncRNAs were associated with the pathophysiology of numerous disorders. Metastasis-associated lung adenocarcinoma transcript 1 (MALAT1) is a widely expressed lncRNA that was demonstrated to induce damage to micro- and macrovascular damage through alterations in its expression driven by hyperglycemia (De Rosa et al. 2018). Endothelial dysfunction is caused by this lncRNA, which binds one of the proinflammatory ligands known as serum amyloid antigen 3 and promotes the production of ROS, IL-6, and TNF-α. The lncRNA AK081284 is another one that implies the development of cardiomyopathy; it is expressed more during hyperglycemia (Zhang et al. 2018f). By inducing cardiac fibroblasts to produce more collagen and transforming growth factor β1 (TGF-β1), upregulation of AK081284 increases cardiac fibrosis. Diabetic patients exhibit elevated competing endogenous RNA, such as myocardial infarction–associated transcript (MIAT) expression in the myocardium, sponges 3p and miR-22, upregulates DAPK2 production, and promotes cardiomyocyte death (Zhou et al. 2017).

### circRNA involvement in DCM

Heart fibrosis is one of the main causes of diabetic cardiomyopathy. Overexpression of Col1a2, Col3a1, and α-SMA in cardiac fibroblasts has been associated with the overproduction of circRNA_000203 in the myocardium of patients with diabetes (Wan et al. 2021). Furthermore, circRNA_000203 causes the upregulation of genes linked to blocks Col1a2 and CTGF target moiety by sponging miR-26b 5p as well as fibrosis in the myocardial fibroblasts. CircRNA_010567 promotes the excision of fibrosis-associated protein via modulating miR-141 in conjunction with its target gene TGF β1. The development of diabetic cardiomyopathy is mediated by the circRNA_010567/miR-141/TGF β1 mechanism, an essential regulatory system implicated in cardiac fibrosis (Zhou and Yu 2017).

## The epigenetic mechanisms involved in DCM

**Fig. 3:**
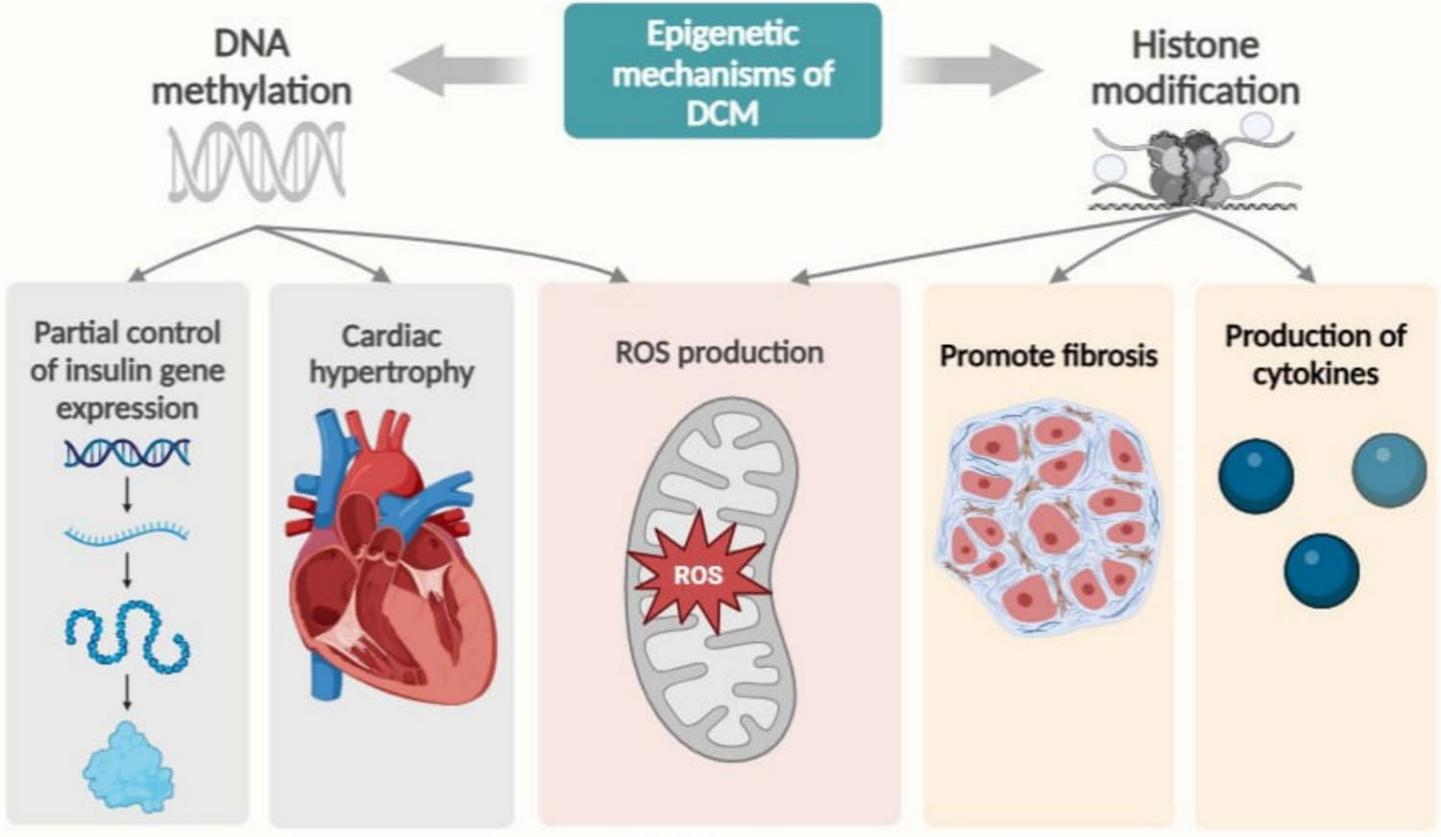
The epigenetic mechanisms involved in diabetic cardiomyopathy (DCM)

The term “epigenetics” describes inherited changes to genes’ function without altering the nucleotide sequence through DNA and chromatin alterations that remain across the cycles of cell division (Singh et al. 2011). Histone acetylation, RNA-based mechanisms, and DNA methylation are common epigenetic processes (De Rosa et al. 2018) as displayed in Fig. 3.

### DNA methylation

The role of epigenetic processes in diabetic cardiomyopathy is poorly understood. However, some available investigation suggests that epigenetics may play a role in the development of diabetic cardiomyopathy. Since the growth of β-islet cells depends on insulin promoter CpG demethylation, DNA methylation partially controls the expression of the insulin gene (Zheng et al. 2017). Disrupted oxidative phosphorylation in the mitochondria and gene silencing have been linked to increased transcriptional co-enhancer peroxisome proliferator–activated receptor (PPAR)γ co-activator-1 α methylation, which acts as a mitochondrial signaling pathways regulator in the pancreatic islets of type 2 diabetic patients (Bogdarina et al. 2007). TNF-α modulates calcium handling by raising DNA methyltransferase levels, which in turn induces hypermethylation of the promoter of SERCA2a, which reduces SERCA function (Zheng et al. 2017). Cardiac hypertrophy is associated with significant involvement of DNA methylation due to the upregulation of genes implicated in the renin–angiotensin–aldosterone system mechanism caused by undermethylation of the proximal promoter of the AT1b angiotensin receptor (Bogdarina et al. 2007). According to Tate et al. (2017) investigation, oxidative damage driven by ROS in diabetic patients results in the apoptosis of cardiac cells through p53-dependent stimulation. This cascade leads to involves the de novo methylation of p53-inducible p21WAF1/CIP1 (whose protein inhibits several cyclin-cyclin-dependent kinase complexes) and the progression of diabetic cardiomyopathy (Tate et al. 2017).

### Histone modification

Histone deacetylase 3 (HDAC3) activity is significantly increased in diabetic mice’s hearts, and inhibiting these enzymes blocks the inflammation and oxidative stress driven by diabetes, enhancing cardiac remodeling and mitigating mouse dysfunction (Xiao et al. 2021). Histone changes also impact fibroblast development by triggering pro-fibrotic pathways created by TGF-β and the synthesis of ECM-associated proteins, which in turn promotes fibrosis (Tate et al. 2017). Although there is currently insufficient evidence, studies have linked HDACs, particularly class I HDACs, which are prohypertrophic (Ke et al. 2021). Chronic hyperglycemia in diabetic circumstances causes the aortic vascular endothelial cells to produce ROS, methylate histone H3K4, and activate NF-κB-p65 epigenetically. All of these modifications eventually promote the development of diabetic cardiomyopathy (Deng et al. 2021). Additionally, HDACs play an important role in inflammation associated with diabetes. In vivo investigations have demonstrated elevated recruitment of NF-κB and histone acetylation, along with histone acetyltransferases, at the promoter regions of inflammatory genes, thereby augmenting the release of cytokines in diabetes and suggesting the connection between HDACs and cardiomyopathy and inflammation (Ke et al. 2021).

## Gut microbiota

The development of diabetic cardiomyopathy can be triggered by the metabolites of gut microbiota, which control inflammation, insulin resistance, autophagy, oxidative stress, and apoptosis (Yuan et al. 2022).

### Gut microbiota–mediated oxidative stress

There is ongoing debate on the connection between oxidative stress and gut microbiota. It was reported that the gut epithelial lining can produce oxidative stress at physiological concentrations (Dumitrescu et al. 2018). Increases in lipopolysaccharide (LPS) levels triggered by gram-negative bacteria may result in significant production of ROS, mostly from infiltrating neutrophils and macrophages (Sah et al. 2011). Moreover, it was revealed that TMAO might mediate the inositol-requiring enzyme 1α (IRE1α)/X-box binding protein 1 (XBP-1) mechanism, hence promoting oxidative stress (Zhang and Hu 2020). Nonetheless, some researchers contend that oxidative stress can be lessened by the gut flora. For instance, there was an investigation that demonstrated that *Lactobacillus fermentum* markedly reduced the formation of ROS in 3T3-L1 preadipocytes generated by hydrogen peroxide (H_2_O_2_) (Kumar et al. 2020). However, another study showed that by controlling the production of antioxidant enzymes, *Lactobacillus* Shirota could defend gut cell–like epithelial cells from oxidative and inflammatory stress caused by 2,2′-azobis (2-amidinopropane) dihydrochloride (Finamore et al. 2018). Diversity in the gut microbiota’s composition and richness might account for this paradox. Thus, preserving the stability of the gut microbiota and enhancing the types of host-protective bacteria present in the gut microbiota are of the greatest significance. This will represent a significant advancement in the management of DCM.

### Gut microbiota–mediated insulin resistance

Insulin resistance can be mitigated by a healthy gut flora (Saad et al. 2016). It has been proposed that the gut microbiota’s potential to control metabolism and energy balance is significantly affected by the intake of lipid nutrients, which affect bacterial SCFA production levels (Cani 2014). Furthermore, it has been demonstrated that a dysregulated gut microbiota and a disrupted intestinal barrier can induce elevated levels of secondary Bas, branched-chain amino acids (BCAAs), and LPS synthesis, all of which might contribute to insulin resistance (Saad et al. 2016). SCFA supplementation alleviated insulin resistance and reduced obesity in diet-induced obese mice (Perry et al. 2016). In other investigations, butyric-producing bacteria, including *F. prausnitzii*, utilize discrete sampling of the free fatty acid receptor 2 (FFAR2) to cause colon L cells to release glucagon-like peptide 1 (GLP-1), which lowers insulin resistance (Christiansen et al. 2018). Insulin sensitivity is impacted by the activation of TGR5 by BAs, which stimulates intestinal cells to secrete GLP-1 as well as indirectly influences pancreatic β-cells’ production of insulin (Duboc et al. 2014). To counteract insulin resistance and lessen the harm that diabetes causes, TGR5 may be a crucial target.

### Gut microbiota–mediated inflammation

SCFAs have anti-inflammatory properties. Propionate, acetate, and butyrate are the three primary byproducts of the colon fermentation of fibers that produce SCFAs. Besides, there was a report that showed that butyrate inhibits histone deacetylase (HDAC), which in turn decreases proinflammatory molecules in gut macrophages, such as NO, interleukin-6, and interleukin-12 (Chang et al. 2014). Additionally, it has been demonstrated that propionate greatly lowers cardiovascular damage by decreasing the quantity of effector memory T cells and T-helper 17 cells (Bartolomaeus et al. 2019).

Nevertheless, the gut microbiota and its bacterial byproducts have both pro- and anti-inflammatory properties. For instance, it was proposed that TMAO-induced inflammation may activate the inflammasome ROS-thioredoxin interacting protein (TXNIP)-NLRP3, which in turn could lead to endothelial dysfunction in the endothelial cells of umbilical vein of humans (Sun et al. 2016). Similarly, it was demonstrated that TMAO can trigger the production of IL-1β and interleukin (IL)−18, two inflammatory cytokines, in response to NLRP3 inflammation (Yue et al. 2017). By activating the mitogen-activated protein kinase (MAPK) and NF-κB signaling mechanism, TMAO significantly raises inflammatory markers, including E-selectin, ICAM1, IL-6, and cyclooxygenase-2 (COX-2), which in turn induces vascular inflammation (Seldin et al. 2016). This paradox might be explained by variations in the gut microbiota’s combination. The inflammatory response is widely recognized as a key pathogenic mechanism of DCM. All of the evidence suggests that altering the gut microbiota’s composition to boost the number of anti-inflammatory bacteria might lead to novel treatments for DCM.

### Gut microbiota–mediated apoptosis and pyroptosis

The study by Saito et al. (2019) found that Shiga toxin–induced HT29 cell death was prevented by *Bacteroides fragilis* (*B. fragilis*) (Saito et al. 2019). However, gut microbiomes also possess anti-apoptotic properties. Additionally, it was demonstrated that BIF-mediated Caco-2 cell death was triggered by TNF-α (Nie et al. 2020). According to Li and Elsasser’s (2005) study, butyric acid–induced renal epithelial cells undergo apoptosis and cell cycle arrest (Li and Elsasser 2005).

Pyroptosis is a kind of proinflammatory cell death that is accompanied by cytokine production, cell swelling, and damage to subcellular organelles (Liu et al. 2019b). According to data, TMAO causes vascular endothelial cells to undergo pyroptosis by generating reactive oxygen species (ROS), which ultimately results in atherosclerosis (Liu et al. 2019b). Furthermore, *Vibrio proteolyticus* from borers’ guts caused pyroptosis by triggering caspase-1 and the NLRP3 inflammasome, which in turn caused the release of IL-1β. On the other hand, another study showed that sodium butyrate shields glomerular endothelial cells from damage driven by elevated glucose levels by having an antipyroptotic action (Gu et al. 2019).

## Current Treatment Options

The current treatment options available for DCM encompass both pharmacological intervention and lifestyle modification strategies tailored to improve patient outcomes (Moka et al. 2024). The pharmacological interventions available in this regard include a range of agents such as antiatherosclerosis medications, antihypertensive drugs, anti-diabetic agents, and specific agents aimed at addressing chronic heart failure (Table 2). Several different medications have been shown to significantly improve symptoms and also contribute to the reduction of cardiovascular events among patients suffering from T2DM (Arnold et al. 2020). Intensive management of blood glucose levels has been associated with a marked decrease in the cumulative incidence of heart failure, particularly in overweight patients who benefit from metformin therapy (Pandey et al. 2020).

**Table 2:** Current pharmacological treatment options for diabetic cardiomyopathy (DCM)

Drug name	Typical dosage	Mode of action in DCM	Common side effects	Role in DCM	Monotherapy/combination therapy	References (human studies)
SGLT2 inhibitors (e.g., empagliflozin, dapagliflozin)	5–25 mg/day	Improves cardiac energy metabolism, reduces cardiac fibrosis and inflammation	Genitourinary infections, dehydration, hypotension	Primary	Both	Wiviott et al. (2019)
GLP-1 receptor agonists (e.g., liraglutide)	0.6–1.8 mg/day (SC)	Enhances insulin sensitivity, reduces cardiac oxidative stress and apoptosis	Nausea, vomiting, pancreatitis (rare)	Primary	Both	Marso et al. (2016)
ACE inhibitors (e.g., lisinopril, enalapril)	5–40 mg/day	Inhibits RAAS, reduces myocardial fibrosis and hypertrophy	Cough, hyperkalemia, hypotension	Primary	Both	Packer et al. (1999), Zhong et al. (2023)
ARBs (e.g., losartan, valsartan)	50–100 mg/day	Blocks angiotensin II, reducing inflammation and fibrosis	Dizziness, hyperkalemia, angioedema	Primary	Both	McMurray et al. (2014)
Beta-blockers (e.g., bisoprolol, carvedilol)	1.25–25 mg/day	Reduces sympathetic overactivation, prevents cardiac remodeling	Fatigue, bradycardia, dizziness	Primary	Both	Di Lenarda et al. (2004)
Mineralocorticoid receptor antagonists (e.g., spironolactone)	25–50 mg/day	Reduces myocardial fibrosis and sodium retention	Hyperkalemia, gynecomastia, renal dysfunction	Adjunctive	Combination	Zannad et al. (2011)
Metformin	500–2000 mg/day	Improves insulin sensitivity, reduces oxidative stress and inflammation	Gastrointestinal upset, lactic acidosis (rare)	Adjunctive	Both	Radzioch et al. (2024)
Statins (e.g., atorvastatin)	10–80 mg/day	Anti-inflammatory reduces oxidative stress and endothelial dysfunction	Muscle pain, liver enzyme abnormalities	Adjunctive	Both	Mills et al. (2011)
DPP-4 inhibitors (e.g., sitagliptin)	100 mg/day	Enhances GLP-1 levels, reduces oxidative stress and fibrosis	Nasopharyngitis, headache	Adjunctive	Both	Liu et al. (2019a)
PPAR-γ agonists (e.g., pioglitazone)	15–45 mg/day	Weight gain, edema, heart failure risk	Improves insulin sensitivity, reduces inflammation and fibrosis	Adjunctive	Both	Wilding (2012)
Natriuretic peptides (e.g., sacubitril/valsartan) (Entresto®)	24/26 mg to 97/103 mg twice daily	Hypotension, hyperkalemia	Enhances natriuresis, reduces fibrosis and inflammation	Adjunctive	Combination	Sauer et al. (2019)
Diuretics (e.g., furosemide)	20–80 mg/day	Electrolyte imbalance, dehydration	Reduces fluid overload, decreases preload	Adjunctive	Combination	Shah et al. (2004)
Digoxin (Lanoxin®)	0.125–0.25 mg/day	Arrhythmia, nausea	Increases myocardial contractility, reduces neurohormonal activation	Adjunctive	Combination	Gheorghiade et al. (2004)
Ivabradine	5–7.5 mg twice daily	Bradycardia, luminous phenomena	Inhibits HCN channels. Channels reduce heart rate without affecting contractility	Adjunctive	Monotherapy	Ide et al. (2019)

All references provided are based on human studies; no animal-based studies were included

*DCM* diabetic cardiomyopathy, *SGLT2* sodium-glucose cotransporter 2, *GLP-1* glucagon-like peptide-1, *ACE* angiotensin-converting enzyme, *ARBs* angiotensin II receptor blockers, *RAAS* renin–angiotensin–aldosterone system, *HCN* hyperpolarization-activated cyclic nucleotide-gated

Furthermore, a comprehensive meta-analysis has indicated that metformin therapy is associated with a significant reduction in the risk of experiencing a composite endpoint of all-cause mortality, myocardial infarction, and stroke when compared with the administration of sulfonylurea-based therapy (Monami et al. 2021).

More recently, a retrospective cohort study has illuminated the fact that T2DM patients, regardless of whether they also presented with heart failure, experienced a markedly reduced risk of all-cause mortality and hospitalization for heart failure when treated with metformin, in stark contrast to those receiving sulfonylureas as their therapeutic (Zhang et al. 2024a).

In the current landscape, the treatment regimen for DCM indeed incorporates essential lifestyle modifications alongside the critical management of hyperglycemia and diabetes-induced complications such as chronic inflammation, oxidative stress, myocardial fibrosis, and other associated complications (Galis et al. 2024). Research on the most effective anti-diabetic therapies continues to focus on those treatments that specifically target T2DM patients, both with and without the presence of heart failure. Within this context, the treatments that have demonstrated the highest efficacy in T2DM patients concurrently managing heart failure include sodium-glucose cotransporter 2 inhibitors as well as glucagon-like peptide-1 receptor agonists (Scheen 2020). Clinicians and healthcare providers have the option to select from a variety of medications that are proven to be effective in lowering blood glucose levels, enhancing insulin sensitivity, or providing organ protection in order to improve the clinical symptoms associated with DCM prominently. However, since personalized medicine should be integrated with the latest treatment options and the progression of DCM is complex, chronic, and slow, some patients may need intensive intervention strategies. Recently, natural compounds have been proposed to improve metabolic disorders, ameliorate myocardial injury, and ameliorate DCM progression. This review discusses the recent research on natural compounds used to treat DCM.

## Future directions in the management of DCM

Since the management of diabetic cardiomyopathy is still in the process of being fully established, many different strategies might be selected for future research directions and/or innovative treatments. Personalized medicine, which focuses on tailoring treatment to the individual characteristics of each patient, may serve as a particularly effective approach to treating patients who appear resistant to conventional pharmacological therapy due to unique alterations in downstream signaling pathways within each individual (Sugandh et al. 2023). As advancements in medical science progress, it is anticipated that not only the gene expression profiles but also the genetic variability of patients, combined with the comprehensive profiling of expressed microRNAs (miRNAs), will increasingly become the central focus of personalized molecular therapy tailored specifically to the needs of diabetic cardiomyopathy patients (Schiano et al. 2020). Additionally, the emerging field of nutrigenetics holds significant promise for identifying new and potentially more effective drug options (Shaman 2024). Exploring the treatment of diabetic cardiomyopathy through methods that extend beyond traditional hypoglycemic agents represents a vital area for future research. Establishing an association with these agents or even considering the substitution of them with certain natural compounds that possess cardioprotective potential is a rational strategy for clinical implementation.

In conclusion, the development of combination therapies that incorporate both natural compounds and new pharmaceutical drugs can highlight and facilitate personalized treatment in the management of diabetic cardiomyopathy. This approach could successfully counteract the deleterious effects of diabetes on the heart and may be beneficial in determining the optimal patient-specific dosage of antihyperglycemic agents to prevent any subsequent damage to the cardiovascular system that might otherwise occur. Technologies could shed light on the in vivo mechanism of DCM. Thus, non-invasive methods based on digital technologies and imaging could be useful for the early diagnosis and monitoring of DCM. New diagnostic modalities and therapeutic targets need to be uncovered, and advances in omics are expected. Such advances may lead to the development of much-needed animal models to help unravel disease mechanisms and identify novel pathways for intervention. Industry-academic partnerships would accelerate these discoveries and provide potential therapies for DCM. Continuous educational initiatives, lobbying efforts, and, most importantly, communication and awareness are essential to translate more promising treatments and interventions and develop guidelines in the future to improve DCM management strategies (Yasmin et al. 2021; Achenbach et al. 2022).

## Natural compounds interacting with one or more targets and evidence supporting their use in the management of DCM

Natural products remain a prospective source of scaffolds with a broad variety of bioactivity and structural diversity that could be employed directly in drug development or as a basis for improving new medication(Atanasov et al. 2021). Recent clinical trials and in vitro and in vivo studies have demonstrated the effectiveness of natural ingredients as anti-diabetic medicines (Alam et al. 2018; Jugran et al. 2021). New drugs with high efficacy, few side effects, and low cost are needed in light of the need for clinical treatment and scientific research on DCM. Natural products have been discussed extensively for their therapeutic effects, indicating their great potential for treating DCM (Yao et al. 2024).

The use of natural products for DCM intervention has been the subject of numerous studies over the last ten years, but the significance of these findings still needs to be investigated and resolved (Yao et al. 2024).

### 11-Keto-β-boswellic acid (AKBA)

11-Keto-β-boswellic acid (AKBA) is a triterpenoid derived from *Boswellia serrata*. The main cardioprotective effects of AKBA are mediated by activation of the AMPK pathway, activation of the Nrf2 antioxidant axis, and inhibition of the NF-κB inflammatory cytokine axis. Together, these pathways enhance mitochondrial function, inhibit oxidative stress, and prevent apoptosis. AKBA functionally improves systolic and diastolic cardiac performance, reduces oxidative damage, decreases inflammatory cytokine levels, and improves mitochondrial efficiency, all of which preserve myocardial structure (AlTamimi et al. 2023).

### 4-O-methylhonokiol

4-O-methylhonokiol, a biflavonoid isolated from Magnolia bark, has as its primary cardioprotective mechanism the activation of the AMPK-mediated lipid metabolism pathway. The activation of this pathway improves lipid metabolism, decreases oxidative stress, and attenuates myocardial fibrosis, suggesting that this pathway may be useful in the treatment of both metabolic and structural cardiac damage in DCM (Zheng et al. 2019).

### Aloe-emodin derivative

The cardioprotective effects of Aloe-emodin, an anthraquinone derivative from *Aloe vera*, were shown to be due to inhibition of the NLRP3 inflammasome pathway. This inhibition prevents inflammatory cell death and structural damage in the myocardium by reducing myocardial inflammation and pyroptosis (Hu et al. 2023).

### Asiaticoside

The cardioprotective effects of asiaticoside, a triterpenoid saponin from *Centella asiatica*, are mediated by activation of the AMPK/Nrf2 pathway that increases antioxidant defenses, decreases oxidative stress, and promotes autophagy. Collectively, these actions result in improved cardiac function, reduced myocardial damage, and improved cellular homeostasis (Xu et al. 2024).

### Bakuchiol

Bakuchiol, a monoterpene derived from the seeds of *Psoralea corylifolia*, activates the SIRT1/Nrf2 signaling pathway to reduce oxidative stress and inflammation. These mechanisms prevent myocardial fibrosis, reduce oxidative damage, and decrease cardiac hypertrophy, with overall myocardial structure and function preserved (Ma et al. 2020).

### Cinnamic acid

Cinnamic acid, a phenolic acid derived from cinnamon, inhibits cytokines, such as TNF-α, IL-6, and IL-1β, and has anti-inflammatory effects. Also, it helps improve insulin sensitivity and treats dyslipidemia. These effects prevent cardiac hypertrophy, lower serum lipid levels, and attenuate systemic inflammation (Nair et al. 2022).

### Cinnamon polyphenols

Another derivative of cinnamon, cinnamon polyphenols, regulates cardiac energy metabolism by modulating the mTOR/PGC1α signaling pathway. This mechanism restores autophagic homeostasis, improves energy metabolism, and reduces oxidative stress, thereby improving myocardial efficiency and cardiac function (Zhang et al. 2024b).

### Costunolide

The cardioprotective effects of costunolide, a sesquiterpene lactone from *Saussurea lappa*, are accomplished primarily through NF-κB and p38-MAPK inhibition and Nrf2 activation. Together, these mechanisms reduce myocardial fibrosis, improve myocardial function, and attenuate oxidative stress. Costunolide protects cardiac tissue from DCM by targeting inflammation and oxidative damage (Jin et al. 2023).

### Cyclovirobuxine D

Cyclovirobuxine D (CVB-D), an alkaloid derived from *Buxus microphylla*, demonstrated cardioprotective effects by inhibition of the NLRP3 inflammasome and prevention of pyroptosis. This inhibition decreases myocardial damage, fibrosis, and inflammation-mediated cell death. CVB-D also provides a unique therapeutic strategy to mitigate inflammatory damage in DCM by targeting pyroptosis (Gao et al. 2022a).

### Daphnetin

A coumarin derivative from *Daphne* genus plants, daphnetin, inhibits the mitogen-activated protein kinase (MAPK) pathway, suppresses the endoplasmic reticulum (ER) stress, and demonstrates anti-inflammatory and anti-apoptotic properties. These mechanisms inhibit myocardial fibrosis, dampen inflammation, and block ER stress–induced apoptosis. The therapeutic relevance of daphnetin resides in its targeted approach to inflammation and cellular stress pathways in DCM (Zhao et al. 2024).

### Ellagic acid

Ellagic acid, a phenolic acid found in nuts, berries, and pomegranates, has cardioprotective actions that involve the activation of the SIRT1 pathway and suppression of NF-κB signaling. These actions decrease inflammatory cytokine levels, prevent myocardial fibrosis, and reduce oxidative stress. Its cardioprotective potential is highlighted by the compound’s ability to preserve myocardial function and attenuate fibrotic remodeling (Altamimi et al. 2020).

### Eugenol

Eugenol, a phenol derived from the clove tree, has anti-inflammatory and anti-fibrotic effects by reducing TNF-α, TGF-β, caspase 3, vascular endothelial growth factor A (VEGF-A), and collagen IV levels. It also increases SOD activity and thereby reduces oxidative stress. Combined effects reduce hyperglycemia-induced myocardial damage and improve cardiac function (Qar et al. 2022).

### Gastrodin

Gastrodin, Phenolic glycoside extracted from *Gastrodia elata*, activates the Nrf2 pathway (Gao et al. 2022b) through GSK-3β signaling, restores the levels of GSH, decreases the levels of oxidative stress, protects from apoptosis, and attenuates cardiomyocyte toxicity (Dong et al. 2021). The compound has the potential to manage cellular oxidative damage and preserve cardiomyocyte viability in hyperglycemic settings (Gao et al. 2022b).

### Notoginsenoside R1

Notoginsenoside R1, a saponin derived from *Panax notoginseng*, activates the Akt/Nrf2 signaling pathway and inhibits the TGF-β/Smad pathway. These actions together decrease oxidative stress, prevent apoptosis, and attenuate myocardial fibrosis (Zhang et al. 2018a).

### Oxymatrine

An alkaloid from *Sophora flavescens*, oxymatrine, modulates the SIRT1/AMPK signaling pathway and inhibits the TGF-β pathway. Oxidative stress, mitochondrial dysfunction, and cardiac fibrosis are also reduced by these mechanisms. The dual ability of oxymatrine to modulate oxidative and inflammatory pathways suggests its therapeutic potential in treating structural and metabolic abnormalities in DCM (Seksaria et al. 2023).

### Phlorizin

Phlorizin, a dihydrochalcone extracted from apple peels, regulates cardiac lipid metabolism, modulates mitochondrial function, and attenuates AGEs. Together, these mechanisms all diminish lipid accumulation, oxidative stress, and mitochondrial dysfunction. Preserving mitochondrial integrity and preventing lipotoxic damage, phlorizin also improves myocardial structure and cardiac function (Cai et al. 2013).

### Protocatechuic acid

Protocatechuic acid, a phenolic acid extracted from *Sansevieria roxburghiana*, activates the PI3K/Akt/AMPK pathway while inhibiting NF-κB and PKC pathways. These actions improve glucose metabolism, reduce oxidative stress and inflammation, and preserve heart histology. Protocatechuic acid has therapeutic relevance because it engages in both metabolic and inflammatory pathways (Bhattacharjee et al. 2017).

### Schisandrin B

A lignan extract of *Schisandra chinensis*, Schisandrin B, inhibits the MyD88-dependent TLR4 signaling pathway, suppresses NF-κB, and blocks the TAK1/MAPK cascade. They reduce inflammation, prevent fibrosis, and improve cardiac function (Luo et al. 2022).

### Sophocarpine

Sophocarpine, a quinolizidine alkaloid from *Sophora flavescens*, inhibits the NF-κB signaling pathway that drives inflammation in DCM. It also attenuates mitochondrial dysfunction, inflammation, and cardiomyocyte apoptosis. Its ability to suppress chronic inflammation and mitochondrial damage makes it a potential therapeutic for preventing structural and functional myocardial damage in diabetic hearts (Zou et al. 2019a).

### Syringaresinol

Cardioprotection was exerted by syringaresinol, a lignan from cereals and medicinal plants, through downregulation of TGF-β, fibronectin, α-SMA, Smad2/3, Bax/Bcl2, and Keap1 signaling pathways. These molecular mechanisms lead to reduced myocardial hypertrophy and fibrosis and enhanced cardiac structure and function. Syringaresinol provides a multi-target approach to managing DCM by simultaneously addressing oxidative stress, inflammation, and fibrosis (Li et al. 2020).

### Syringic acid

The effects of syringic acid are mainly by reducing oxidative stress and promoting mitochondrial biogenesis. Lipid peroxidation and protein carbonylation are important markers of oxidative damage, and syringic acid lowers both. The protective mechanisms provided by syringic acid enhance myocardial health in general and reduce oxidative damage; therefore, syringic acid is a promising therapeutic agent in the management of DCM (Sabahi et al. 2021).

Based on their structural properties, 80 other natural compounds reported for DCM were found and categorized into six groups: flavonoids, terpenoids, alkaloids, quinones, glycosides, and others (Table 3).

**Table 3:**
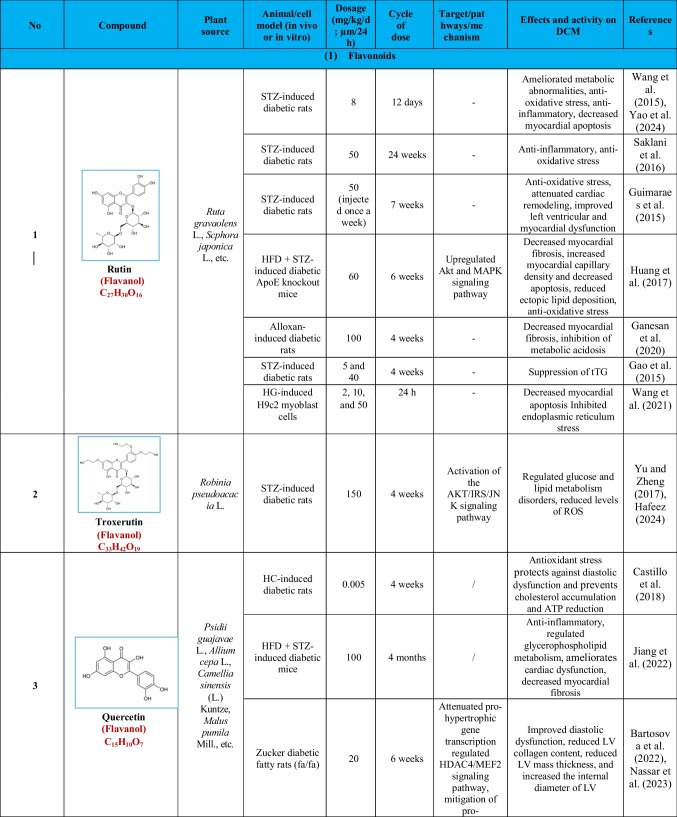

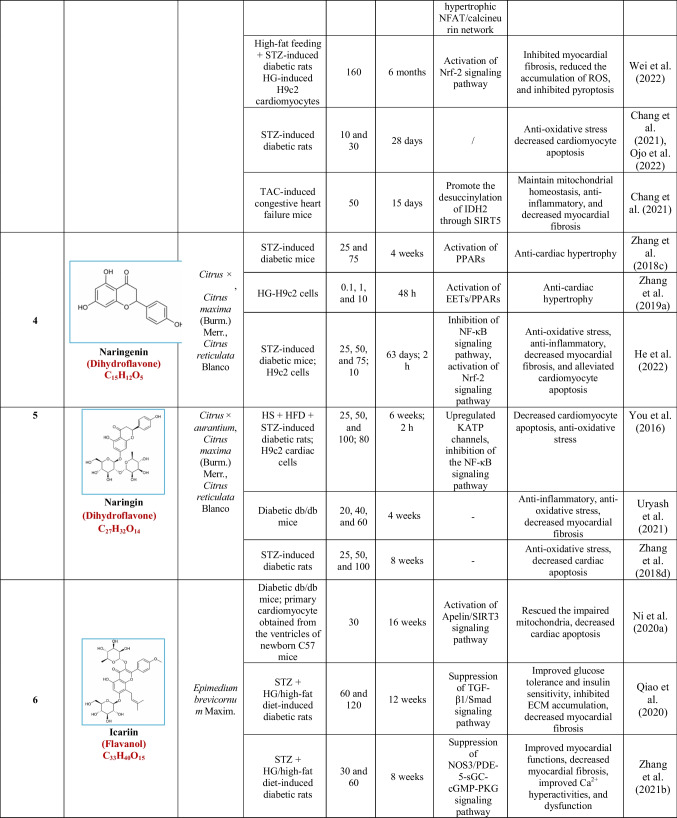

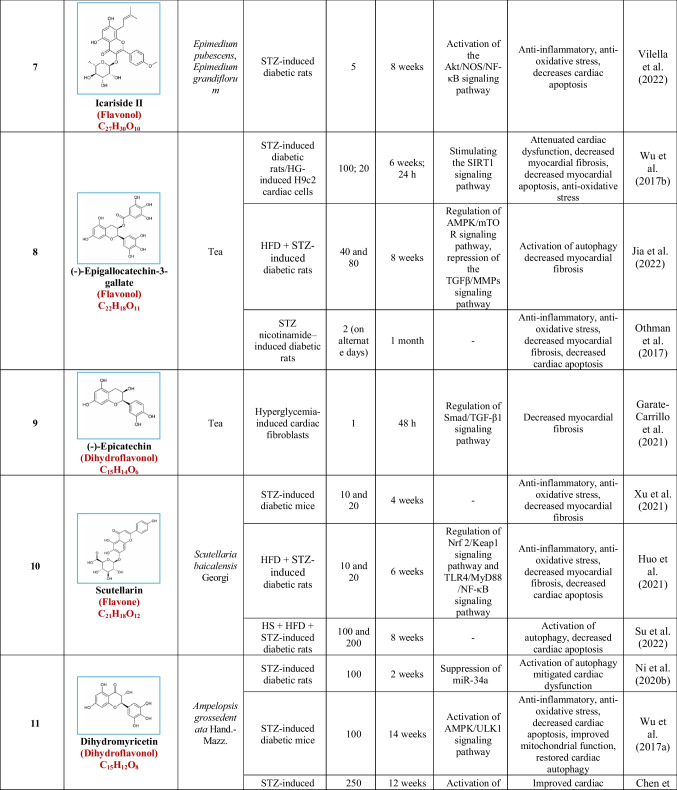

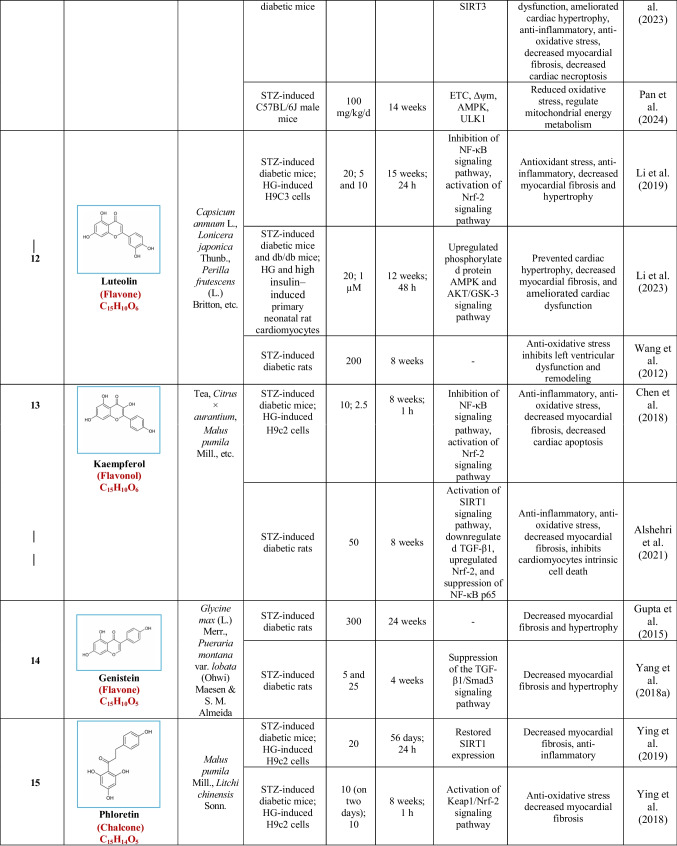

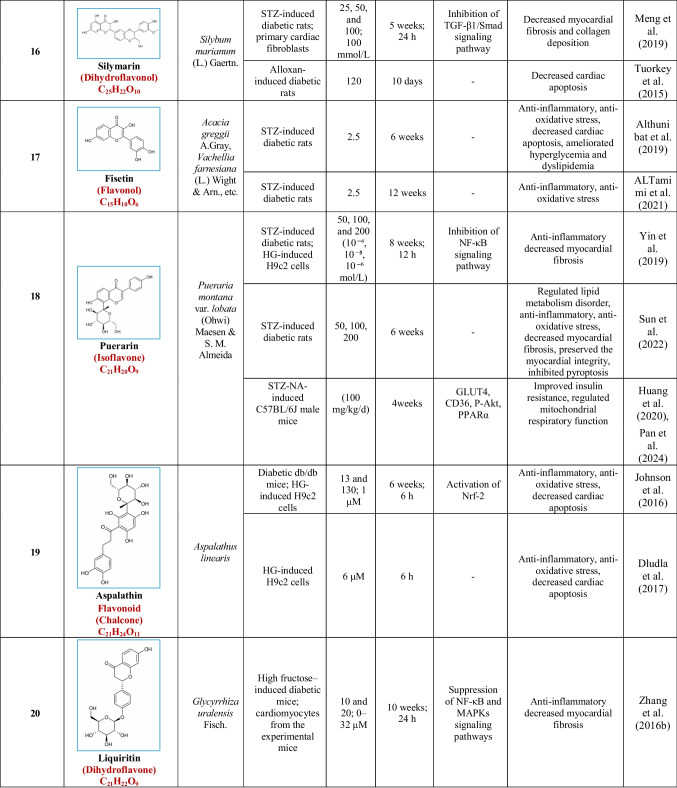

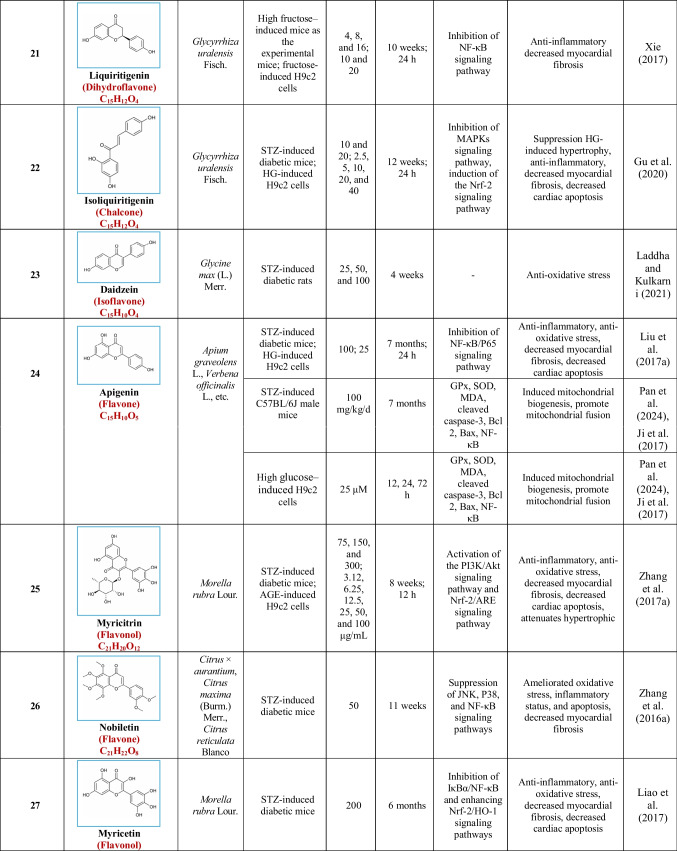

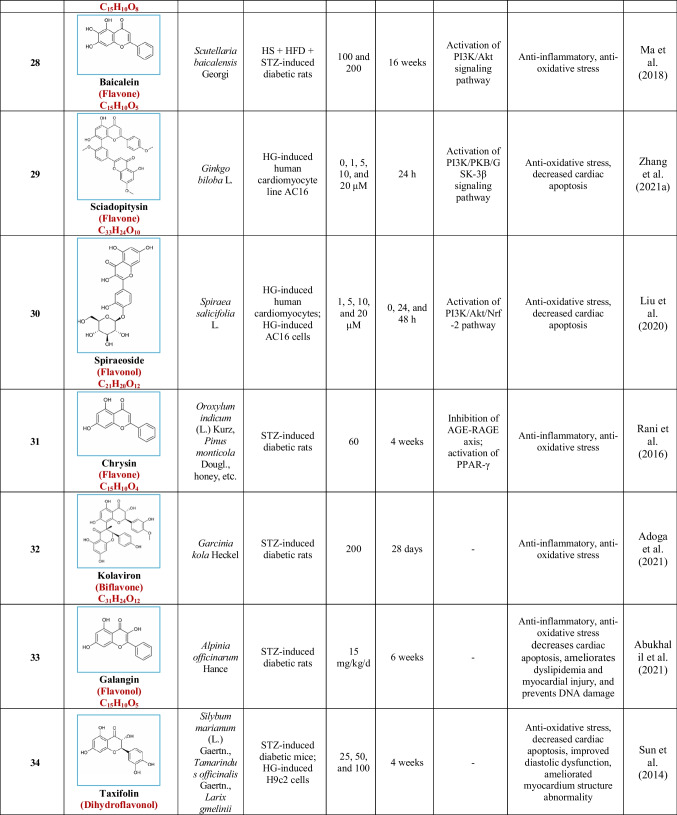

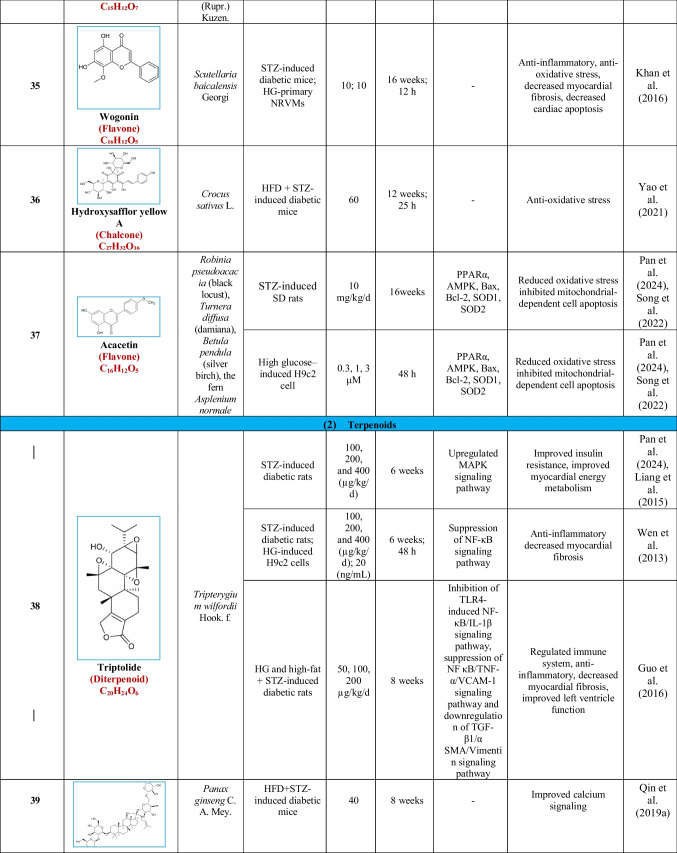

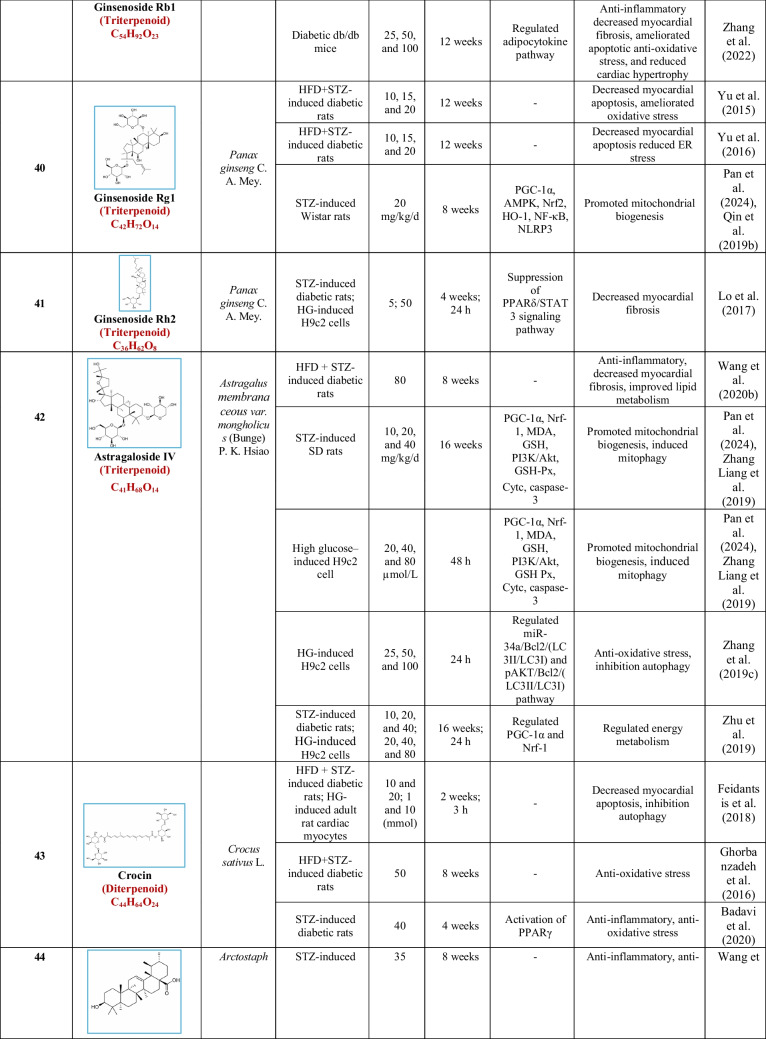

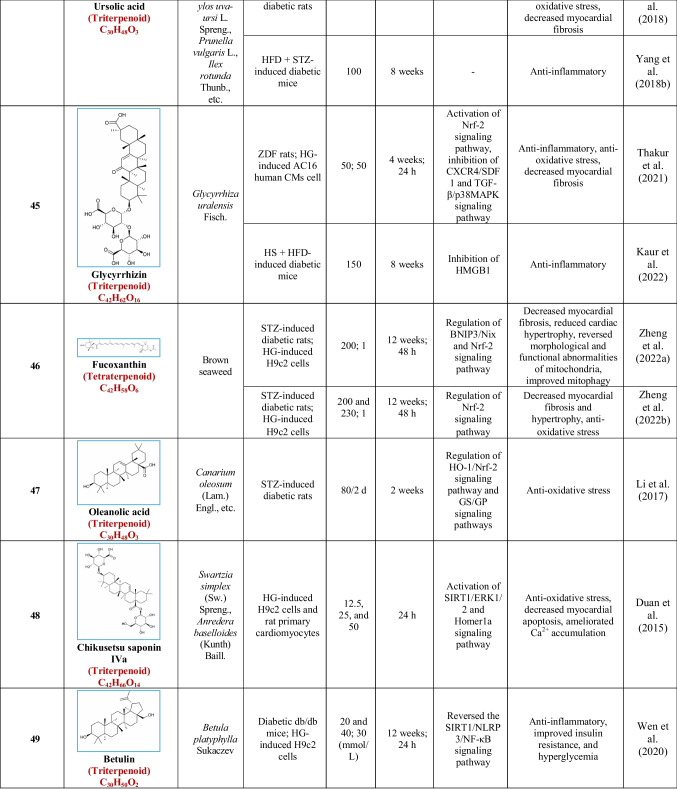

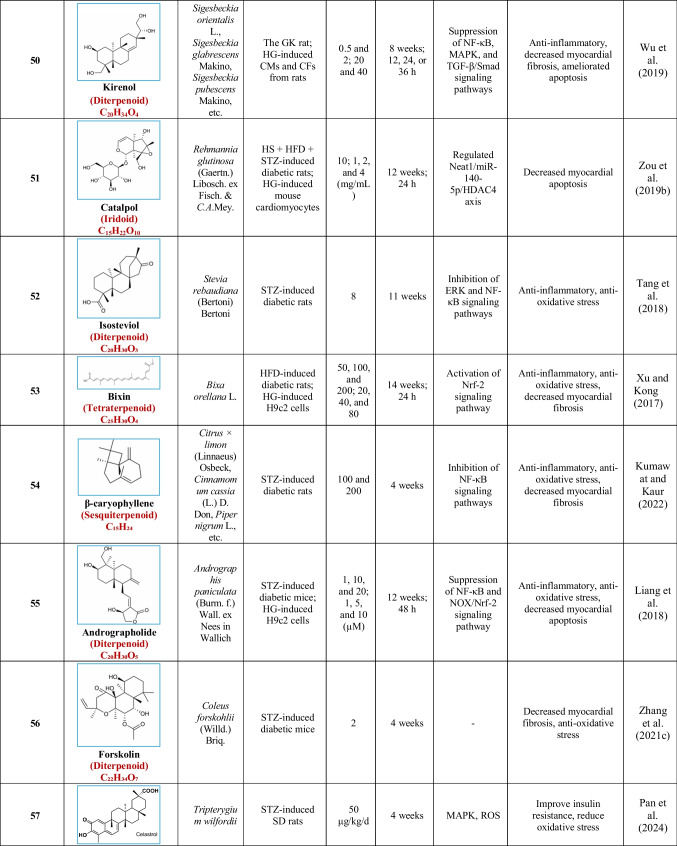

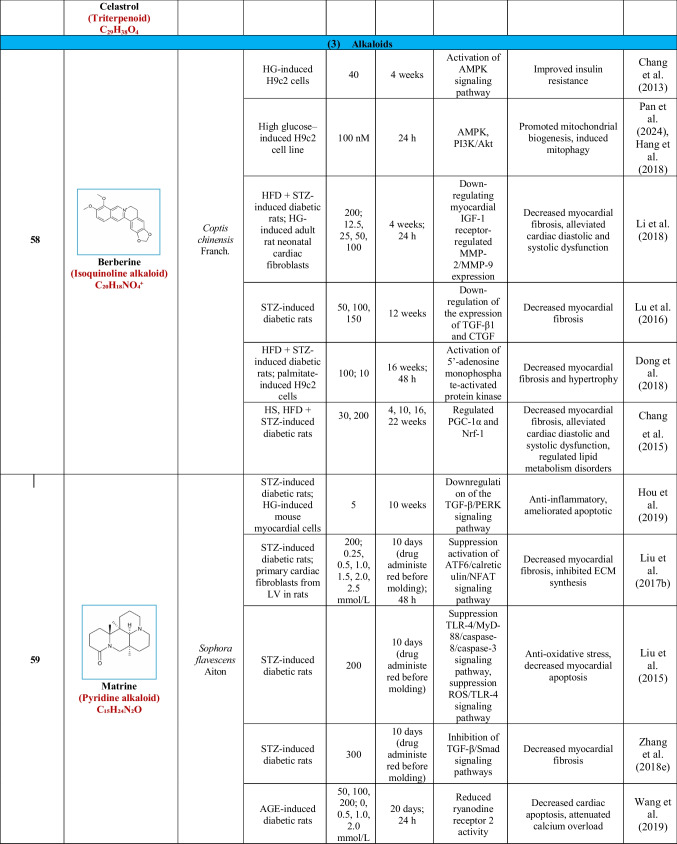

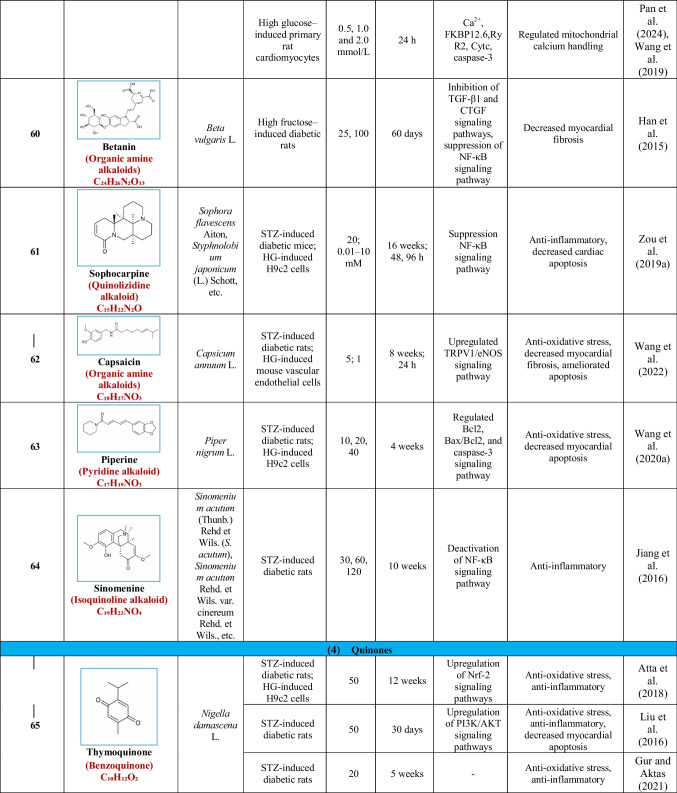

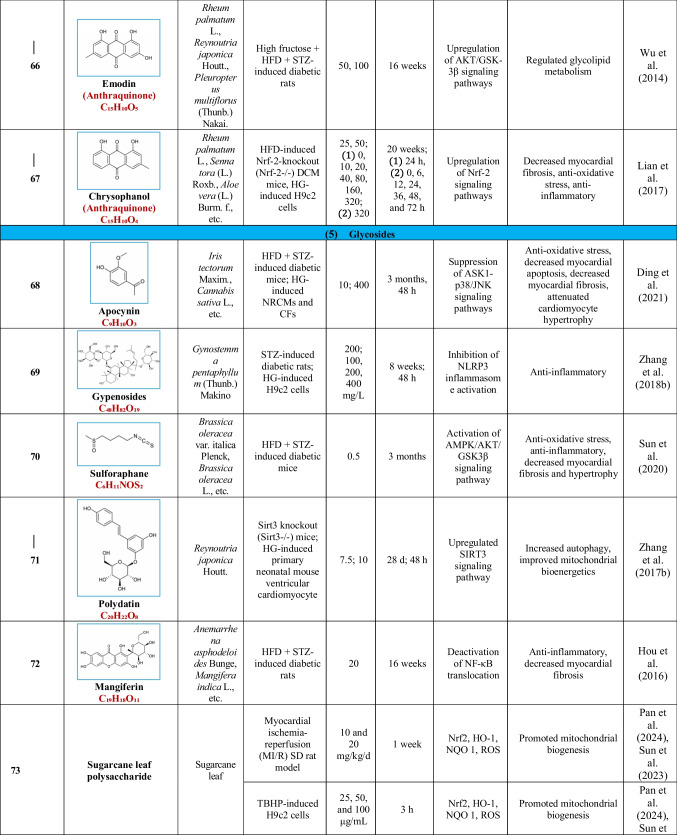

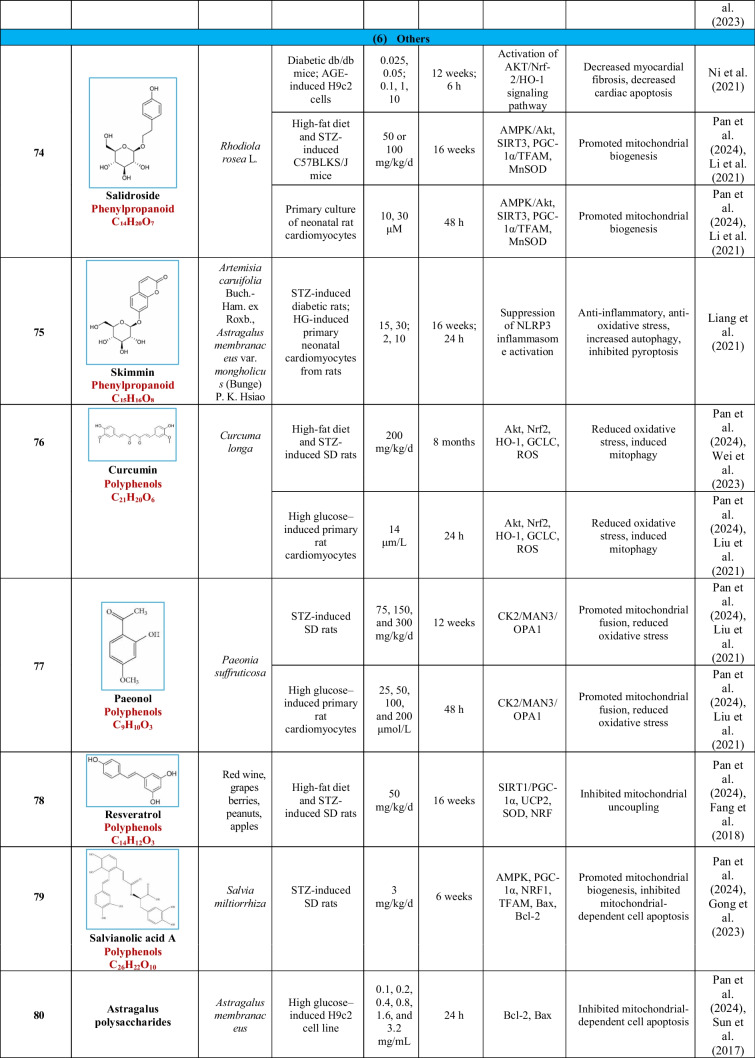
Natural compounds interacting with one or more targets and evidence supporting their use in the management of DCM

## Conclusions

DCM is a complex disorder characterized by structural and functional abnormalities in the heart that are primarily associated with diabetes. Extensive preclinical research has focused on discovering molecular targets that help it develop. Although various drugs have been produced for DCM, many have serious adverse effects and have even been removed from the market. As a result, there is an increased interest in natural product–based therapy. Epidemiological studies indicate that natural products may help reduce the incidence of chronic diseases. Natural products’ efficacy in treating DCM depends on their antioxidant and anti-inflammatory properties, regulation of cell death (apoptosis, necroptosis, pyroptosis, and autophagy), regulation of glucose and lipid metabolism, regulation of Ca^2+^ homeostasis, anti-fibrosis, and protection of mitochondria and ER function and structure. Natural product research in animal models has shown promise for controlling DCM; nevertheless, there is still a fundamental challenge: many medicinal plants lack strong scientific data and clinical validation. To solve this issue, clinical trials are required to assess the efficacy, toxicity, dose, bioavailability, and absorption of natural therapies. A thorough understanding of how natural compounds target DCM, as well as improved screening methodologies and insights into structure–activity connections, will be critical for progressing drug development in this field.

## Data Availability

All source data for this work (or generated in this study) are available upon reasonable request.

## Abbreviations

ACE: Angiotensin-converting enzyme
AGEs: Advanced glycation end-products
AKT: Protein kinase B
AMP: Adenosine monophosphate
AMPK: AMP-Activated protein kinase
ATP: Adenosine triphosphate
α-SMA: Alpha-smooth muscle actin
Bax: Bcl-2-associated X protein
BCAAs: Branched-chain amino acids
Bcl-2: B-cell lymphoma 2
BIF: Bifidobacterium
BP: Blood pressure
Ca^2+^: Calcium ions
CHF: Congestive heart failure
COX-2: Cyclooxygenase-2
CD36: Cluster of differentiation 36
CRP: C-reactive protein
CVB-D: Cyclovirobuxine D
CV: Cardiovascular
CVD: Cardiovascular diseases
DAG: Diacylglycerol
DCM: Diabetic cardiomyopathy
DM: Diabetes mellitus
ER: Endoplasmic reticulum
ECM: Extracellular matrix
FFAR2: Free fatty acid receptor 2
FFAs: Free fatty acids
FOXO: Forkhead box O
FOXO1: Forkhead box O1
GLP-1: Glucagon-like peptide 1
GLUT: Glucose transporter
GLUT1: Glucose transporter type 1
GLUT3: Glucose transporter type 3
GLUT4: Glucose transporter type 4
GSH: Glutathione
GSK-3β: Glycogen synthase kinase-3 beta
GSDMD: Gasdermin D
H2S: Hydrogen sulfide
H3K4: Histone 3 lysine 4
H9c2: Rat cardiomyoblast cell line
HbA1c: Glycated hemoglobin
HDAC: Histone deacetylase
HFD: High-fat diet
HO-1: Heme oxygenase-1
IκBα: Inhibitor of nuclear factor kappa B alpha
IL-1β: Interleukin-1 beta
IL-6: Interleukin-6
IL-12: Interleukin-12
IL-18: Interleukin-18
ICAM1: Intercellular adhesion molecule 1
Keap1: Kelch-like ECH-associated protein 1
LGE: Late gadolinium enhancement
lncRNA: Long non-coding RNA
LPS: Lipopolysaccharides
MALAT1: Metastasis-associated lung adenocarcinoma transcript 1
MAPK: Mitogen-activated protein kinase
MIAT: Myocardial infarction–associated transcript
miRNA: MicroRNA
MRI: Magnetic resonance imaging
mTOR: Mechanistic target of rapamycin
MyD88: Myeloid differentiation primary response 88
NFAT: Nuclear factor of activated T-cells
NF-κB: Nuclear factor kappa B
NLRP3: NOD-like receptor pyrin domain-containing protein 3
NO: Nitric oxide
NOX: NADPH oxidase
NLRP3: NOD-, LRR- and pyrin domain-containing protein 3
Nrf2: Nuclear factor erythroid 2-related factor 2
NT-proBNP: N-terminal pro-B-type natriuretic peptide
PGC1α: Peroxisome proliferator–activated receptor gamma co-activator 1-alpha
PGE2: Prostaglandin E2
PI3K: Phosphatidylinositol-3-kinase
PKA: Protein kinase A
PKB: Protein kinase B
PKC: Protein kinase C
PRKCG: Protein kinase C gamma
RAAS: Renin–angiotensin–aldosterone system
ROS: Reactive oxygen species
SCFAs: Short-chain fatty acids
SERCA: Sarco/endoplasmic reticulum Ca^2+^-ATPase
SERCA2a: Sarco/endoplasmic reticulum calcium-ATPase 2a
SR: Sarcoplasmic reticulum
SIRT1: Sirtuin 1
SIRT3: Sirtuin 3
Smad2/3: Sma- and Mad-related protein 2/3
SOD: Superoxide dismutase
STZ: Streptozotocin
T2DM: Type 2 diabetes mellitus
TMA: Trimethylamine
TMAO: Trimethylamine N-oxide
TAK1: Transforming growth factor beta-activated kinase 1
TGF-β: Transforming growth factor beta
TLR4: Toll-like receptor 4
TNF-α: Tumor necrosis factor alpha
TXNIP: Thioredoxin interacting protein
ULK1: Unc-51-like autophagy activating kinase 1
VEGF-A: Vascular endothelial growth factor A
XBP-1: X-box binding protein 1
